# A Retrospective Study Analyzing the Appropriateness of Initial Treatment of *Clostridium difficile* in Patients with Active Malignancy

**DOI:** 10.1155/2018/7192728

**Published:** 2018-05-27

**Authors:** Aaron Fisher, Pradeep Khanal, Ewa Gniado, Leila Khaddour, Molly Orosey, Ismail Hader, Siddhartha Yadav, Alexandra Halalau

**Affiliations:** ^1^Beaumont Health, Royal Oak, MI, USA; ^2^Bronson Methodist Hospital, Kalamazoo, MI, USA; ^3^University of Cincinnati Medical Center, Cincinnati, OH, USA; ^4^Henry Ford Hospital, Detroit, MI, USA; ^5^Oakland University William Beaumont School of Medicine, Rochester, MI, USA; ^6^Mayo Clinic, Rochester, MN, USA

## Abstract

**Background:**

*Clostridium difficile* infection (CDI) is the leading cause of hospital-associated gastrointestinal illness. Previous studies reported that patients with active malignancy are at high risk for CDIs, and yet they are still classified as nonsevere CDI and initially treated with metronidazole. Our aim is to investigate the need for the escalation of antibiotic therapy in patients with CDI and active cancer treated with oral metronidazole versus oral vancomycin.

**Methods:**

This is a retrospective study of adult patients admitted with CDI and any underlying active malignancy at Beaumont Hospital, Royal Oak, Michigan, from January 2008 to December 2014. Inclusion criteria included age > 18 years old, polymerase chain reaction- (PCR-) proven CDI, and active malignancy.

**Results:**

197 patients were included in the final analysis. 44.8% of the metronidazole group required escalation of therapy compared to 15.2% in the vancomycin group (*p* value = 0.001). 29.8% of the combination group (metronidazole and vancomycin) underwent deescalation of antibiotics, which was significantly higher compared to 2.2% of patients in the vancomycin group (*p* value < 0.001).

**Discussion:**

Our results support the initial use of vancomycin or a combination (metronidazole and vancomycin) versus metronidazole in patients with CDI and active malignancy.

## 1. Introduction

*Clostridium difficile* infection (CDI) is the leading cause of hospital-associated gastrointestinal illness and has been reported to be more prevalent, more severe, more refractory to standard therapy, and more likely to relapse than previously described [1, 2]. The annual cost for diagnosis, management, and treatment of CDI in the United States is approximately 3.2 billion dollars [3]. Since the early 2000s, CDI has been on the rise both in the hospital and throughout the community [4]. In 2011, there was approximately 453,000 cases of CDI throughout the United States and roughly 29,300 patients died secondary to CDI. An estimated 159,700 cases of CDI were community acquired compared to 107,600 which were hospital acquired [5].

Patients with active malignancy have been considered high risk for developing CDI, as studies have reported that up to 7% of patients receiving chemotherapy will develop CDI and 8.2% may develop severe enterocolitis [6, 7]. Furthermore, Chopra et al. reported up to a 9-fold increase in CDI risk in hospitalized hematopoietic stem cell transplant recipients versus the general public [8]. In Parmar et al., metronidazole was noted to have low cure rates for patients with CDI and hematologic malignancy. Also, the associated morbidity and mortality of these patients is much higher than the general population [9].

Currently, there is no formal definition to determine the severity of CDI. A few different scoring systems have been used. One of the most widely used and convenient scoring systems from the European Society of Clinical Microbiology and Infectious Disease indicates severe versus nonsevere CDI if one of the following are present: white blood cell count is greater than or equal to 15,000 cells/mm^3^, serum albumin < 3 g/dl, and/or serum creatinine level is greater than or equal to 1.5 times the premorbid level [10]. Yet, many patients with active cancer may have neutropenia which would possibly underestimate their disease severity.

According to the European Society of Clinical Microbiology and Infectious Disease, oral metronidazole 500 mg three times daily for ten days is the recommended initial antibiotic treatment for nonsevere CDI (strength of recommendation—A, quality of evidence—I). Oral vancomycin 125 mg four times daily for 10 days is the recommended initial antibiotic for severe CDI (strength of recommendation—A, quality of evidence—I) [10].

Our hypothesis is based on the assumption that patients with active malignancy might be predisposed to a more severe clinical CDI which might not be responsive to single therapy with oral metronidazole.

## 2. Methods

### 2.1. Study Design

The study was a retrospective cohort designed to compare the need for escalation of initial CDI therapy in patients with *Clostridium difficile* colitis and active malignancy treated with oral metronidazole or oral vancomycin. Institutional review board approval was obtained before initiation of the study.

### 2.2. Setting

The patients were identified through a query to the electronic health record at Beaumont Health (BH), Michigan. BH is the largest healthcare system serving patients across southeastern Michigan, including the greater Detroit area and has three hospitals in Royal Oak, Troy, and Grosse Pointe.

### 2.3. Participants

The patient data was collected from January 1, 2008 to December 31, 2014. BH's electronic health record (EHR; EPIC system, Verona, WI, USA) was queried between the study dates to identify patients meeting the inclusion criteria: age greater than 18 years old, documented CDI, and active malignancy. Exclusion criteria include patients that elected hospice care. Chart review and analysis of each eligible patient was performed to determine whether the patients had active malignancy, which was defined as patients treated with radiation, chemotherapy, or surgery within six months prior to the diagnosis of CDI.

**Figure 1:**
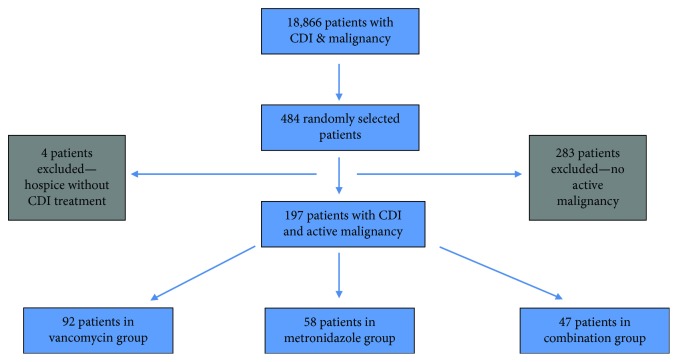
Patient selection flowsheet.

The International Classification of Diseases, Ninth Revision, Clinical Modification (ICD-9-CM) codes were used to identify all adult patients with both malignancy and documented CDI (18,866 patients). From all 18,866 patients, a computer-generated program randomly selected 484 patients in order to obtain a final sample size of at least 75 in each group, per the sample size calculation. All the 484 patient charts were manually reviewed and 283 patients were excluded as they did not meet our criteria for active malignancy. 4 other patients were excluded as they enrolled in hospice and did not receive CDI treatment. The remaining 197 patients were further divided into groups based on the treatment they received for CDI. The groups included metronidazole (58 patients), vancomycin (92 patients), and a combination of both metronidazole and vancomycin (47 patients) (Figure 1).

### 2.4. Outcomes

Our primary objective was to investigate the need for escalation of antibiotic therapy in patients with active malignancy and CDI, initially treated with either oral metronidazole or oral vancomycin. Appropriate initial antibiotic use was considered if there was no escalation of antibiotic treatment throughout the hospital course (i.e., metronidazole to vancomycin or a combination). Our secondary objectives included deescalation of antibiotic therapy, length of stay, all-cause in-hospital mortality, 90-day all-cause mortality, and 90-day recurrence rate.

### 2.5. Variables

The variables that were assessed included patient age, gender, race, severity of CDI, sepsis, type of malignancy, malignancy staging, and the treatment method for active cancer. Severe CDI was determined if one of the following were present: (1) white blood cell count is greater than or equal to 15,000 cells/mm^3^, (2) serum albumin < 3 g/dl, and/or (3) serum creatinine level greater than or equal to 1.5 times the premorbid level. Severity of CDI was considered unknown if not all diagnostic criteria were available. Sepsis was determined if two of the following were present: (1) temperature greater than 100.4 or less than 95 degrees Fahrenheit, (2) respiration rate greater than 20 breaths per minute, (3) heart rate greater than 90 beats per minute, and/or (4) white blood cell count greater than 12,000 cells/mm^3^ or less than 4000 cells/mm^3^. Malignancy type was reported into five categories including: colorectal, gastrointestinal other than colorectal, solid malignancy other than gastrointestinal, hematologic, and other (which refers to either multiple primary cancers or unknown primary). Malignancy was further categorized according to the stage and included local/regional, metastatic, hematologic, and unknown. Treatment of malignancy included chemotherapy, radiation, and surgery. Individual chart analysis was performed to determine whether patients required escalation of antibiotics. Escalation of antibiotics was defined as patients who were initially treated with metronidazole and were either changed to vancomycin or required the addition of vancomycin or patients initially treated with vancomycin who required the addition of metronidazole. Deescalation of antibiotics was defined as patients who were initially started on a combination, and were transitioned to vancomycin or metronidazole, or started on vancomycin, and transitioned to metronidazole.

### 2.6. Data Source

The initial source of the data was through reports that were extracted from the electronic medical records EPIC through Toad Data Point. A thorough chart review of electronic medical records was also performed to ensure inclusion and exclusion criteria and complete data collection.

### 2.7. Bias

We tried to minimize the potential impact of selection bias in our study by randomly selecting the final sample size from all the patients identified to meet the inclusion criteria. Regular meetings with the data collectors were done throughout the study in order to minimize the information bias. Researcher bias was limited via strict adherence to the research protocol. Standardized protocols for data collection were implemented to minimize the interobserver variability in-between the six data collectors. The possible confounding impact of the *Clostridium difficile* carrier state was not assessed as the data was not available in the chart.

### 2.8. Study Size

Target sample size was determined prior to the start of data collection after consultation with a biostatistician. A sample size of at least 75 in each group was calculated to achieve a power of 81%, with an alpha of <0.05 to detect a 22% difference in the primary outcome in-between the two groups (metronidazole and vancomycin group).

### 2.9. Statistical Analysis

Statistical analysis was performed using SPSS 21 (released in 2012; IBM SPSS Statistics for Windows, Version 21.0, IBM Corp., Armonk, NY). Descriptive statistics were reported as frequencies along with proportions for categorical variables. Means (with two standard deviations), medians, and range were used to describe continuous variables. Fisher's exact test was used to compare categorical variables. For continuous variables, one-way ANOVA was used to compare means and the Mann–Whitney *U*-test was used to compare medians. The Bonferroni method was used to compare column proportions. All tests were two sided. Statistical significance was considered at *p* < 0.05.

## 3. Results

A total of 201 patients out of the 484 patients randomly selected from the total sample of patients were identified through a chart review to have CDI and active malignancy. Four patients, who did not receive treatment for *Clostridium difficile*, were excluded from the study as they were enrolled into hospice. The remaining 197 patients were included in the final analysis. We decided to run this interim statistical analysis which became the final statistical analysis, even though we did not reach the sample size calculated in the metronidazole group (only 58 out of 75 patients), because all of the patients' charts that were randomly selected from the total sample were reviewed. Median age for the entire CDI and active malignancy population was 71 (range 24–96). 70% were Caucasians and 58.4% females. 52.8% of patients had severe CDI and 42.1% had sepsis upon CDI diagnosis. 25% of the patients had GI malignancies, from which 48.8% of them had colorectal cancer. Other solid malignancies accounted for 46.4% of the total patients, while hematologic malignancies were 27%. Of those with solid malignancies, 33% had local disease, 61% had metastatic disease (43.9% of total patients), and 7% had unknown staging. 67.5% of patients were receiving chemotherapy and 23.4% were receiving radiation therapy only.

The patients were divided into 3 groups based on the initial antibiotic use: 58 patients in the metronidazole group (29%), 92 patients in the vancomycin group (47%), and 47 patients who received a combination therapy with vancomycin and metronidazole (24%). The median age of patients in the metronidazole group was 73.5 (range 24–94) compared to 67.0 (range 33–96) in the vancomycin group and 71.0 (range 28–87) in the combination group. In the metronidazole group, 63.8% were females and 74.1% were Caucasians compared to 57.6% female and 68.5% Caucasians in the vancomycin group, and 53.2% female and 68.1% Caucasians in the combination group. In the metronidazole group, 58.6% of the patients had severe CDI compared to 43.5% in the vancomycin group and 63.8% in the combination group. In the metronidazole group, 0.0% had nonsevere CDI compared to 7.6% in the vancomycin group and 8.5% in the combination group. In regard to treatment differences according to malignancy type, of the patients in the metronidazole group, 8.6% had colorectal cancer compared to 10.9% in the vancomycin group and 19.6% in combination. In the metronidazole group, 13.8% had gastrointestinal (GI) malignancy other than colorectal cancer compared to 10.9% in the vancomycin group and 15.2% in the combination group. In the metronidazole group, 44.8% had solid malignancies other than GI compared to 54.3% in the vancomycin group and 32.6% in the combination group. In the metronidazole, vancomycin, and combination groups, 63.8%, 72.8%, and 61.7%, respectively, were on chemotherapy, 19.0%, 30.4%, and 14.9%, respectively, were on radiation, and 34.5%, 33.7%, and 38.3%, respectively, received surgery (Table 1).

32.6% of patients with severe CDI were started on metronidazole compared to 38.5% who were started on vancomycin and 28.9% who were started on the combination therapy. The initial treatment of CDI patients with sepsis was done as follows: 24.1% of patients with sepsis were treated with metronidazole compared to 48.2% who were treated with vancomycin and 27.7% who were treated with a combination. In patients with colorectal cancer, the initial choice of treatment was with metronidazole in 20.8% of patients compared to vancomycin in 41.7% and the combination in 37.5%. In the patients with GI malignancy other than colorectal cancer, metronidazole was started in 32.0% of patients compared to vancomycin in 40% of patients and the combination in 28.0% of patients. In the patients with metastatic disease, 30.2% of them were started on metronidazole, while 48.8% were started on vancomycin and 21.0% were started on the combination. From patients receiving chemotherapy, 27.8% of patients were initially treated with metronidazole versus 50.4% with vancomycin and 21.8% with the combination of metronidazole and vancomycin.

### 3.1. Outcomes

**Figure 2:**
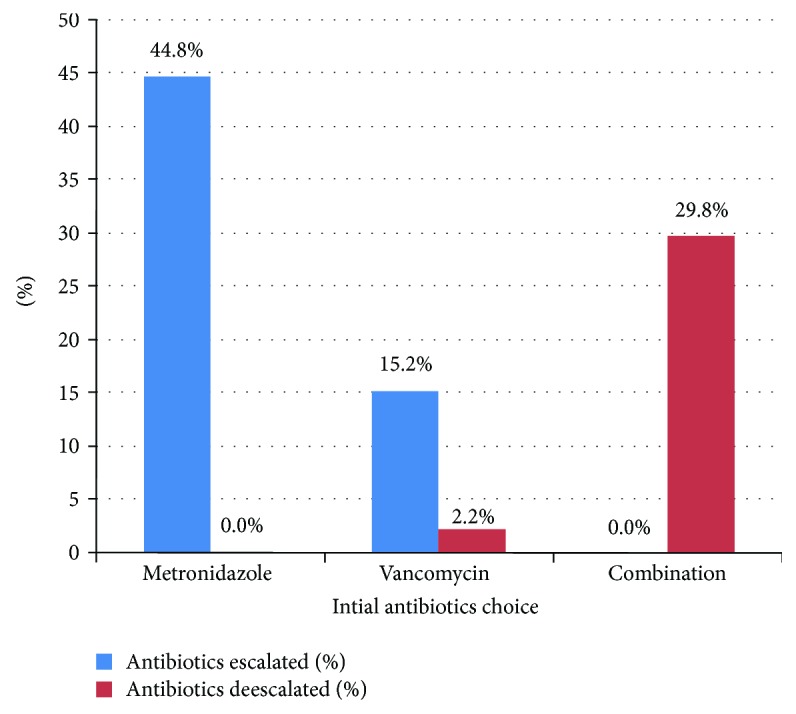
Escalation or deescalation of initial antibiotics.

Escalation of therapy rate was significantly higher in the metronidazole group, which was needed in 44.8% compared to 15.2% in the vancomycin group (*p* value = 0.001). 29.8% of the combination group underwent deescalation of antibiotics, which was significantly higher compared to 2.2% of patients in the vancomycin group (*p* value < 0.001). 55.2% of the patients who were initially started on metronidazole compared to the 84.8% who were initially started on vancomycin were considered to have received an appropriate initial treatment as they did not require escalation of antibiotics (Table 2 and Figure 2).

In-hospital length of stay, in-hospital all-cause mortality rate, and 90-day all-cause mortality rate in all treatment groups are presented in Table 3. It was noted that the 90-day recurrence rate was the highest in the metronidazole group, 23.5%, compared to 23.1% and 11.9% in the vancomycin and combination groups, respectively (*p* value 0.18) (Table 3).

In univariate analysis, age, sex, race, type of malignancy, stage of malignancy, antibiotics choice, and severity of CDI were not associated with 90-day recurrence rates. When these variables were analyzed in the binomial logistic regression model, none of the variables was significantly associated with 90-day recurrence rates as well.

## 4. Discussion

Our study found a significant difference in the need to escalate antibiotic therapy in patients with active malignancy and CDI who were initially started on single therapy with metronidazole when compared to patients initially started on single therapy with vancomycin. Our findings suggest that we may be undertreating this severe disease in patients with active malignancy and are consistent with prior evidence. After a thorough literature search, we could only find one similar study of 79 oncology patients treated for an initial episode of CDI, in which 31.6% of patients who started on oral metronidazole required escalation of therapy [11].

Although our study was not designed to assess the accuracy of current CDI severity classification in patients with active malignancy, our findings suggest that the scoring system from the European Society of Clinical Microbiology and Infectious Disease might underestimate the severity of infection in patients with laboratory abnormalities related to their active malignancies or to their associated treatments. Another widely used and recognized scoring system is from the American College of Gastroenterology that classifies CDI into mild, moderate, and severe. Mild disease is defined as CDI with diarrhea as the only symptom. Moderate disease is defined as CDI with diarrhea but without additional symptoms/signs meeting the definition of severe or complicated CDI below. Severe disease is CDI that presents with or develops during the course of the disease with hypoalbuminemia (serum albumin < 3 g/dl) and either of the following: (1) a white blood cell (WBC) count ≥ 15,000 cells/mm^3^ or (2) abdominal tenderness [4]. This scoring system would also underestimate the severity of the CDI in patients with active malignancy.

A significantly higher percentage of patients who were started on combination therapy underwent deescalation of antibiotics compared to patients who were started on vancomycin alone (29.8% versus 2.2%, *p* value < 0.001). Currently, there are no other studies that have looked into the deescalation of antibiotics in CDI in patients with active malignancy. One explanation for this finding is that practitioners may feel more comfortable deescalating to vancomycin from a combination versus deescalating to metronidazole alone. Alternatively, initial combination therapy may be more efficient at controlling the symptomatology thus giving physicians the confidence to deescalate to single therapy. This is not an example of inappropriate treatment. In our opinion, this is an example of an alternate more aggressive treatment that leads to rapid symptomatic improvement, in which setting the antibiotic deescalation would be considered required and appropriate. We suggest that a deescalation strategy should be considered and recommended in order to prevent the emergence of resistant CDIs.

The current study compared the outcomes between patients with active malignancy and CDI treated with metronidazole, oral vancomycin, or a combination of oral metronidazole and oral vancomycin. More than half (52.8%) of the patients had severe infection and 42.1% of the patients met the criteria for sepsis which is significantly higher than in a similar study by Finn et al. where only 18% had severe infection [11]. Furthermore, in 41.6% of the patients, the severity of CDI could not be determined due to the lack of available diagnostic data. If all these patients had severe CDI, the total percent of patients with severe CDI could have been as high as 94.4%. These findings support the newer CDI statements regarding the fact that recent CDI infections have been reported to be more frequent, more severe, and more refractory to treatment. Thus, there is a need for better diagnostics, early recognition, improved methods to manage severe disease and relapsing disease, and greater attention to infection control and antibiotic resistance [1].

The majority (58.6%) of patients who received metronidazole as their initial antibiotic choice had severe CDI. If all of the patients who were classified in the unknown CDI severity had severe disease, then 100% of the patients in the metronidazole group would have had severe CDI. So it can be argued that these patients should have been treated with oral vancomycin or a combination in the first place. However, the findings of other similar studies also suggest that metronidazole alone may not be the optimal treatment of CDI in oncologic patients [11]. In two large double-blind trials, the cure rates of CDI were significantly lower among oncologic patients compared to those without cancer (*n* = 922, 79.2% versus 88.6%) [12]. Historically, metronidazole has been as effective as oral vancomycin in treating mild to moderate CDI, even in oncologic patients [7]. However, recent observational studies have suggested that metronidazole might be becoming less effective with failure rates from 22% to 26% [13, 14]. Current clinical guidelines use leukocytosis > 15,000 cells/mm^3^ and hypoalbuminemia <3 gm/dl as the marker of severity. Since many patients with malignancy are leukopenic, these guidelines may underestimate CDI severity in the oncologic population [15].

Fecal microbiota transplantation (FMT) has been employed in patients with severe and recurrent CDI who have failed multiple attempts with conventional antibiotic therapy. Several small observational studies have demonstrated the efficacy of FMT in the treatment of CDI in patients with recurrent disease after failed initial antibiotic therapy [16–18]. In a meta-analysis of eleven studies with a total of 273 CDI patients treated with FMT for recurrent CDI, 245 patients experienced clinical resolution with no reported adverse events [19]. Yet, the safety and efficacy of this method has not been well established in the immunocompromised patients. In Hefazi et al., 23 oncologic patients received FMT for recurrent CDI. 8 of the 23 patients had received chemotherapy 12 weeks prior to FMT administration. 18 of the 23 patients were reported to have clinical CDI recovery. The results of this study demonstrate that FMT is a highly effective and safe therapeutic option for recurrent CDI in oncologic patients treated with cytotoxic chemotherapy [20]. However, to our knowledge, there is no known data investigating FMT as the initial treatment modality for oncologic patients.

Our 90-day recurrence rate and 90-day all-cause mortality rates were 19.8% and 25.4%, respectively, which were similar to other studies in oncology patients [9]. The ninety-day recurrence rate was lower in the combination group compared to the metronidazole group (23.5% versus 11.9%, resp.), although it did not reach a level of statistical significance. Finn et al. also demonstrated a higher 90-day retreatment rate in the metronidazole group compared to the oral vancomycin group (26% versus 6% *p* = 0.053, resp.) [11]. 90-day all-cause mortality rate was slightly higher in the combination group compared to the metronidazole group (30.5% versus 29.4%, resp.). A possible explanation for this finding is that patients in the combination group were overall more ill. In the combination group compared to the metronidazole group, patients were more likely to have severe CDI (63.8% versus 58.6%, resp.) and sepsis (48.9% versus 34.5%, resp.). Finn et al. did not find any significant difference in the 90-day mortality rate when comparing combination therapy and metronidazole monotherapy (20% versus 21%, resp.) [11].

Patients with colorectal and other gastrointestinal malignancies were more likely to be initiated on combination or vancomycin compared to metronidazole alone. There is no available literature that looked into differences in presentation and initial antibiotic choices of CDI between malignancy types. We attribute this difference to a more severe presentation of CDI in these patient groups or to the fact that the patients in the combination group were sicker or had a more severe underlying malignancy.

### 4.1. Limitations

Our study is limited by unknown confounding factors inherently present in a single-center retrospective chart review analysis. Although our study design was created to minimize confounding, our data is limited to the data available in the electronic medical record at the time of the data collection. Although there was a significantly higher need for escalation of antibiotics in the metronidazole group compared to the vancomycin group, antibiotics could have been escalated for reasons other than poor response to therapy. Another limitation of our study is that it was not designed to assess the reason of antibiotic escalation. The initial antibiotic choice and the escalation choices were at the discretion of each attending physician. There appeared to be a lack of adherence to the current guidelines, yet this was not formally evaluated. For example, we were unable to assess the severity of CDI in roughly 40% of the patients as albumin and baseline creatinine values were not obtained. Thus, it can be assumed that guidelines were not followed for all patients. Deaths and recurrences that might have occurred outside of our institution were not recorded which can affect the findings of our study.

### 4.2. Strengths

The strengths of our study include a large dataset and low-risk bias. This is the largest study to date looking at the CDI treatment escalation in patients with active malignancy and the first study to investigate the deescalation of antibiotics. Even though we did not reach the sample size calculated for each group, as in the metronidazole group we only had 58 out of 75 patients per group, our results showed a statistically significant difference with a lower sample size.

### 4.3. Conclusions

The results of our study suggest the need for a more aggressive initial treatment of the CDI in active malignancy patients with the use of oral vancomycin or combination therapy versus metronidazole alone as the initial choice of treatment. The variability of CDI severity assessment and choice of initial treatment in-between providers suggest the need of a structured algorithm to be implemented for the evaluation and treatment of CDI in patients with active malignancy. Larger studies with a prospective randomized design would be more valid for assessing the appropriateness of the initial antibiotic treatment in patients with CDI and active malignancy. However, given the importance and the implications of this clinical question, we suggest that immediate guideline changes need to be considered in this patient population. Regarding deescalation therapy, we suggest that deescalation should be considered based on the patient's clinical status. The appropriate timing and method of CDI antibiotic treatment deescalation are still unknown and further investigation is warranted.

## Figures and Tables

**Table 1:** Baseline characteristics of patients based on initial antibiotics choice.

	Total *n* = 197	Metronidazole *n* = 58	Vancomycin *n* = 92	Combo *n* = 47	*p* value
Age, median	71.0	73.5	67.0	71.0	0.18
Age, mean	69.9 ± 13.3	71.9 ± 13.8	68.6 ± 13.1	70.2 ± 12.8	0.32
Age, range	24 to 96	24 to 94	33 to 96	28 to 87	
*Gender*					0.54
Female (%)	115 (58.4)	37 (63.8)	53 (57.6)	25 (53.2)	NS
Male (%)	82 (41.6)	21 (36.2)	39 (42.4)	22 (46.8)	NS
*Race*					0.45
Caucasian (%)	138 (70.1)	43 (74.1)	63 (68.5)	32 (68.1)	NS
African American (%)	21 (10.7)	4 (6.9)	13 (14.1)	4 (8.5)	NS
Other (%)	11 (5.6)	1 (1.7)	6 (6.5)	4 (8.5)	NS
Unknown (%)	27 (13.7)	10 (17.2)	10 (10.9)	7 (14.9)	NS
*Severity of CDI*					0.01
Severe CDI (%)	104 (52.8)	34 (58.6)	40 (43.5)	30 (63.8)	NS
Unknown (%)	82 (41.6)	24 (41.4)	45 (48.9)	13 (27.7)	<0.05^#^
Nonsevere CDI (%)	11 (5.6)	0 (0.0)	7 (7.6)	4 (8.5)	NS
*Sepsis*					0.30
Present (%)	83 (42.1)	20 (34.5)	40 (43.5)	23 (48.9)	NS
Absent (%)	114 (57.9)	38 (65.5)	52 (56.5)	24 (51.1)	NS
*Malignancy type*					0.18
Colorectal (%)	24 (12.2)	5 (8.6)	10 (10.9)	9 (19.6)	NS
GI^∗^ other than colorectal (%)	25 (12.8)	8 (13.8)	10 (10.9)	7 (15.2)	NS
Other solid (%)	91 (46.4)	26 (44.8)	50 (54.3)	15 (32.6)	<0.05^#^
Hematologic (%)	53 (27.0)	18 (31.0)	22 (23.9)	13 (28.3)	NS
Other (%)	3 (1.5)	1 (1.7)	0 (0.0)	2 (4.3)	NS
*Malignancy stage*					0.82
Local/regional (%)	46 (23.5)	11 (19.0)	22 (23.9)	13 (28.3)	NS
Metastatic (%)	86 (43.9)	26 (44.8)	42 (45.7)	18 (39.1)	NS
Hematologic (%)	53 (27.0)	18 (31.0)	22 (23.9)	13 (28.3)	NS
Unknown (%)	11 (5.6)	3 (5.2)	6 (6.5)	2 (4.3)	NS
*Chemotherapy*					0.32
Yes (%)	133 (67.5)	37 (63.8)	67 (72.8)	29 (61.7)	NS
No (%)	64 (32.5)	21 (36.2)	25 (27.2)	18 (38.3)	NS
*Radiation*					0.08
Yes (%)	46 (23.4)	11 (19.0)	28 (30.4)	7 (14.9)	NS
No (%)	151 (76.6)	47 (81.0)	64 (69.6)	40 (85.1)	NS
*Surgery*					0.85
Yes (%)	69 (35.0)	20 (34.5)	31 (33.7)	18 (38.3)	NS
No (%)	128 (65.0)	38 (65.5)	61 (66.3)	29 (61.7)	NS

^∗^Gastrointestinal. ^#^There is a statistical significance between the vancomycin group and the combination group. NS: not significant at the 0.05 level between the 3 groups.

**Table 2:** Treatment efficacy.

	Metronidazole as initial antibiotic (*n* = 58)	Vancomycin as initial antibiotic (*n* = 92)	Combination as initial antibiotic (*n* = 47)	*p* value
Escalation of antibiotics (%)	26 (44.8)	14 (15.2)	N/A	<0.001
Deescalation of antibiotics (%)	N/A	2 (2.2)	14 (29.8)	<0.001

**Table 3:** Outcomes in all treatment groups.

	Total (*n* = 197)	Metronidazole (*n* = 34)	Vancomycin (*n* = 104)	Combination (*n* = 59)	*p* value
Length of stay, days (median)^∗^	9	9	9	10	0.89
Length of stay, days (mean)	14 ± 13	14 ± 14	14 ± 14	13 ± 10	0.16
Length of stay, days (range)	0 to 70	1 to 59	0 to 79	1 to 50	
In-hospital all-cause mortality^∗∗^ (%)	20 (10.2)	4 (11.8)	7 (6.7)	9 (15.3)	0.17
90-day all-cause mortality^∗∗^ (%)	50 (25.4)	10 (29.4)	22 (21.2)	18 (30.5)	0.33
90-day recurrence rate^∗∗∗^ (%)	39 (19.8)	8 (23.5)	24 (23.1)	7 (11.9)	0.18

^∗^There was no difference in hospital length of stay. ^∗∗^In-hospital all-cause mortality and 90-day all-cause mortality were higher in the combination group, but not found to be statistically significant in-between all treatment groups. ^∗∗∗^90-day recurrence rate was lower in the combination group compared to the vancomycin or metronidazole groups (*p* = 0.18).
